# Salivary Cortisol and Cortisone Can Circumvent Confounding Effects of Oral Contraceptives in the Short Synacthen Test

**DOI:** 10.1210/clinem/dgad763

**Published:** 2024-01-04

**Authors:** Nils Bäcklund, Staffan Lundstedt, Andreas Tornevi, Anna-Carin Wihlbäck, Tommy Olsson, Per Dahlqvist, Göran Brattsand

**Affiliations:** Department of Public Health and Clinical Medicine, Umeå University, 901 87 Umeå, Sweden; Department of Medical Biosciences, Division of Clinical Chemistry, Umeå University, 901 87 Umeå, Sweden; Department of Public Health and Clinical Medicine, Umeå University, 901 87 Umeå, Sweden; Department of Clinical Sciences, Obstetrics and Gynecology, Umeå University, 901 87 Umeå, Sweden; Department of Public Health and Clinical Medicine, Umeå University, 901 87 Umeå, Sweden; Department of Public Health and Clinical Medicine, Umeå University, 901 87 Umeå, Sweden; Department of Medical Biosciences, Division of Clinical Chemistry, Umeå University, 901 87 Umeå, Sweden

**Keywords:** short Synacthen test, salivary cortisol, salivary cortisone, oral contraceptives, adrenal insufficiency, reference limits

## Abstract

**Context:**

Adrenal insufficiency (AI) is usually diagnosed by low plasma cortisol levels following a short Synacthen test (SST). Most plasma cortisol is bound to corticosteroid-binding globulin, which is increased by estrogen in combined estrogen-progestin oral contraceptives (COCs). Women with AI using COCs are therefore at risk of having an apparently normal plasma cortisol level during SST, which would not adequately reflect AI.

**Objective:**

This work aimed to test whether salivary cortisol or cortisone during SST is more robust against the COC effect and to calculate the lower reference limits (LRLs) for these to be used as tentative diagnostic cutoffs to exclude AI.

**Methods:**

Forty-one healthy women on COCs and 46 healthy women without exogenous estrogens underwent an SST with collection of plasma and salivary samples at 0, 30, and 60 minutes after Synacthen injection. The groups were compared using regression analysis with age as covariate and the LRLs were calculated parametrically.

**Results:**

SST-stimulated plasma cortisol levels were significantly higher in the COC group vs controls, while mean salivary cortisol and cortisone levels were slightly lower in the COC group. Importantly, COC use did not significantly alter LRLs for salivary cortisol or cortisone. The smallest LRL difference between groups was seen for salivary cortisone.

**Conclusion:**

Salivary cortisol and especially salivary cortisone are considerably less affected by COC use than plasma cortisol during SST. Due to similar LRLs, a common cutoff for salivary cortisol and cortisone during SST can be used to exclude AI in premenopausal women irrespective of COC use.

Adrenal insufficiency (AI) is a rare condition causing substantial morbidity, increased mortality, and risk of fatal acute adrenal crises (1-3). Timely diagnosis and adequate glucocorticoid replacement therapy is therefore of major importance.

Low plasma cortisol in a Synacthen test is diagnostic for AI (4). However, an increased amount of corticosteroid-binding globulin (CBG) entails a risk for misleadingly normal total plasma cortisol levels as more CBG-bound cortisol will be measured, even though cortisol release and free, biologically active cortisol levels may be low (5-9). Elevated CBG levels are typically seen in hyperestrogenemic states such as pregnancy and with the use of estrogen/progestin-containing combined oral contraceptives (COCs) (10-12). This COC-induced increase of CBG plateaus after just over a week of COC use and weans off 4 to 6 weeks after discontinuation (11, 13-17). CBG induction has been reported to be dependent on the estrogen dose and not to be influenced by the synthetic progestin component of the COC (6, 18). Higher cutoffs for morning plasma cortisol have been suggested for women using COCs (19). However, as the estrogen dose varies between different COC formulations, a common cutoff could be unreliable due to a different degree of CBG induction (6).

Importantly, analyses of salivary cortisol may circumvent COC-induced CBG alterations since only unbound cortisol passes from blood to saliva. Analysis of salivary cortisol has been proposed as an alternative to analyzing plasma free cortisol and has been shown to be less affected by CBG alterations (9, 14, 20-25). However, during passage through the salivary gland, most cortisol is inactivated into cortisone by 11β-hydroxysteroid dehydrogenase type 2 (11β-HSD2) (21, 26). Salivary cortisone has been shown to correlate better with plasma free cortisol than salivary cortisol and to be less susceptible to preanalytical confounders (20, 23, 27, 28). It has previously been shown that salivary samples can be used during the short Synacthen test (SST) in the diagnostic work-up for AI (29-34). Nonetheless, whether salivary cortisol and cortisone levels are unaffected by estrogens during an SST remains to be established together with cutoffs for excluding AI using salivary samples, since previous studies were based on few observations (35-37).

Our hypothesis was that salivary cortisol and cortisone concentrations are not significantly affected by COC use during the SST. In line with this, we wanted to calculate the lower reference limits (LRLs) for plasma cortisol, and salivary cortisol and cortisone during an SST for women with and without the use of COCs.

## Materials and Methods

Eighty-nine women were recruited through advertisements on the Umeå University grounds and Umeå University Hospital, and in social media between 2019 and 2022. Inclusion criteria were women aged 18 to 50 years who were either using monophasic COCs with ethinylestradiol as estrogen (COC group) or were nonusers of estrogens (control group). Exclusion criteria were acute illness, pregnancy, known disease affecting the hypothalamic-pituitary-adrenal axis or thyroid gland, ulcers in the oral cavity, asthma, treated hypertension, licorice consumption during the previous week, and use of any glucocorticoid-containing medications during the previous 4 weeks. No participants used medications known to interfere with the hypothalamic-pituitary-adrenal axis. Women in the control group were allowed to use other contraceptives, including hormonal intrauterine devices if their menstrual cycles were regular. Women in the COC group were included after 7 or more days of active medication, while sampling of women in the control group was performed between days 1 to 12 after the first day of menstruation.

After obtaining written informed consent, an SST was performed in an ambulatory setting between 07:00 and 11:00 hours in a semi-supine position after inserting a peripheral vein catheter in the antecubital vein for blood sampling. Blood and salivary samples were collected simultaneously before (baseline, time 0), and at 30 and 60 minutes after intravenous injection of 250 µg Synacthen (CD Pharmaceuticals AB). Salivary samples were collected using Salivette Cortisol tubes (Sarstedt) by placing the synthetic swab into the mouth and either chewing on it for 60 seconds or placing it under the tongue for 90 seconds for passive absorption before spitting the swab back into the plastic tube to avoid handling and the potential transference of topical hydrocortisone. All Salivette tubes were frozen at −20 °C for at least 24 hours, stored long term at −80 °C, and then thawed and centrifuged (3000*g* for 5 minutes) prior to analysis. Blood was collected into Vacutainer PST II tubes (BD) and plasma was separated to plastic vials after centrifugation at 2000g for 10 minutes.

All plasma and saliva samples were stored at −80 °C for simultaneous analysis to avoid risk of drift in the analytical method. Plasma cortisol was analyzed with Roche Elecsys Cortisol II reagents (Roche catalog No. 07027150190, RRID:AB_3068019) on a Cobas e801 analyzer with a coefficient of variation (CV) of less than 6% at 300 nmol/L. Salivary cortisol and cortisone were analyzed by liquid chromatography–tandem mass spectrometry (LC-MS/MS) with CVs of 6% and 7% for cortisol at levels of 1.5 and 50 nmol/L, respectively, and a CV of 7% for cortisone at both 4 and 110 nmol/L (38). Proficiency testing of the LC-MS/MS method was in accordance with other salivary cortisol and cortisone results in the UK NEQAS Birmingham Quality Specimen Exchange Scheme (distribution 133, October 19, 2022).

The study was approved by the ethics committee of Umeå University (EPN 2018-164-31) and the Swedish Ethical Review Authority (2019-04511).

### Statistics

Plasma cortisol, salivary cortisol, and salivary cortisone data were analyzed with Q-Q plots and the Kolmogorov-Smirnov test, which showed normal distributions within each group. No outliers were identified visually or by using the Dixon method (39). The *t* test was used to test for age difference between the groups. Since the mean age differed between COC users and controls, differences in cortisol and cortisone concentrations between the groups were evaluated using univariate regression with age as covariate. The relative increase of salivary cortisol and salivary cortisone at 30 and 60 minutes was compared to the relative increase of plasma cortisol using the *t* test with Bonferroni correction for multiple comparisons. The effects of different ethinylestradiol doses and different types of synthetic progestins were evaluated using analysis of variance. Reference intervals (RIs) were calculated using the parametric uniformly minimum variance unbiased estimator (UMVUE), and testing for significant difference between LRLs for the COC and control groups were made by bootstrap simulation. A pivot point for 11β-HSD type 2 saturation was estimated using a piecewise linear regression model performed with the “segmented” package in the statistical software R. Analyses were made in IBM SPSS Statistics v27 (IBM Corp), R v4.2.2 (R Core Team), and Microsoft Excel 365 v2212 (Microsoft Corporation) with Analyse-it for Microsoft Excel 6.15 (Analyse-it Software Ltd, http://analyse-it.com/).

## Results

Two women were excluded: one from the COC group due to traces of betamethasone in the salivary samples detected by our LC-MS/MS method and one from the control group as the samples were collected outside the intended phase of the menstrual cycle. The analysis therefore included 41 women using COCs and 46 controls. The COCs used were monophasic containing 0.02 mg (n = 9), 0.03 mg (n = 27), or 0.035 mg (n = 4) ethinylestradiol. The estrogen was combined with different synthetic progestins: 2 mg dienogest (n = 5), 3 mg drospirenone (n = 17), 0.15 mg levonorgestrel (n = 14), or 0.25 mg norgestimate (n = 4) (Supplementary Table S1 (40)). Information about the type of COC used was missing for one participant. Mean (range) age was 27 (19-47) years in the COC group and 32 (20-50) years in the control group (*P* = .014). The time of day for injection of Synacthen did not differ between groups (data not shown). Two plasma samples were missing for one participant in the control group (baseline and 30-minute sample), but all other data from this participant were included. One 60-minute sample was delayed almost 30 minutes due to malfunctioning vein access and this sample was therefore excluded. All other samples were collected within ±7 minutes of the designated time point. No salivary samples had a cortisol:cortisone ratio greater than 1, a test for sample integrity and possible contamination (23, 41).

**Figure 1.**
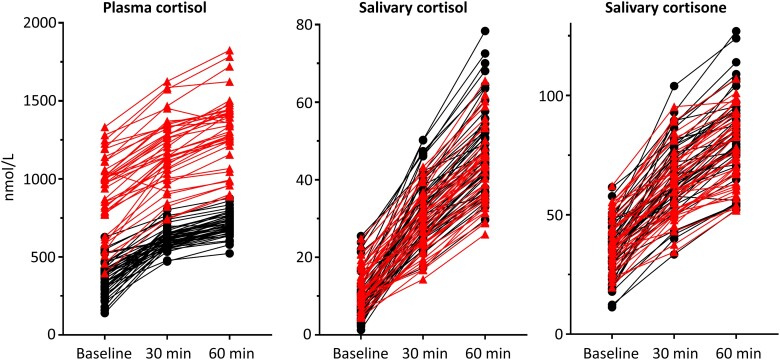
Individual responses to Synacthen at 0, 30, and 60 minutes for plasma cortisol, salivary cortisol, and salivary cortisone. Red triangles indicate participants in the combined oral contraceptives group and black circles indicate those in the control group.

During the SST, the highest concentrations of plasma cortisol, salivary cortisol, and salivary cortisone were found at 60 minutes after Synacthen injection in 85 of the 87 participants. However, for one participant in the COC group, plasma cortisol was highest at baseline and, in another participant in the COC group, salivary cortisone was highest at 30 minutes after Synacthen injection (Fig. 1).

The relative increases in cortisol and cortisone concentrations from baseline to 30 and 60 minutes are shown in Supplementary Table S2 (40). The mean increase of plasma cortisol at 30 and 60 minutes after Synacthen injection was 34% and 51% in the COC group, and 90% and 119% in the control group, respectively. The relative increases of salivary cortisol and salivary cortisone were significantly (*P* < .05 using *t* test with Bonferroni correction) higher than those for plasma cortisol both at 30 and 60 minutes (see Fig. 1 and Supplementary Table S2 (40)).

### Effect of Combined Oral Contraceptives

**Figure 2.**
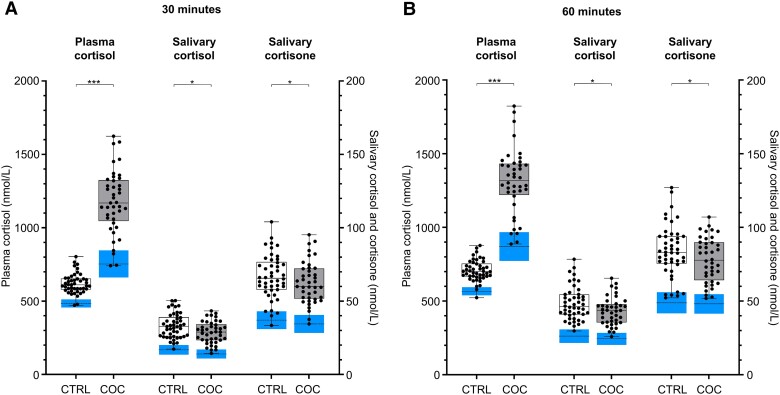
Box plots showing each individual's measurements of plasma cortisol, salivary cortisol, and salivary cortisone following the Synacthen test. Whiskers denote the range, wide dashed black lines denote lower reference limits, and blue boxes around the lower whiskers represent the 90% CIs at A, 30 and B, 60 minutes. Gray boxes indicate participants on combined oral contraceptives (COCs) and white boxes controls (CTRL). *P less than .05; ***P less than .001 for comparison of mean values.

Mean [SD] plasma cortisol levels were significantly higher in the COC group than in the control group at baseline (910 [235] vs 360 [111] nmol/L; *P* < .001), and at 30 minutes (1180 [215] vs 620 [69] nmol/L; *P* < .001) and 60 minutes (1310 [224] vs 707 [71] nmol/L; *P* < .001) after Synacthen injection (Fig. 2). Mean [SD] salivary cortisol levels were slightly but significantly higher in the COC group compared to the control group at baseline (12.5 [5.2] vs 9.1 [5.8] nmol/L; *P* = .048), but lower at 30 minutes (28.7 [7.5] vs 33.1 [8.3] nmol/L; *P* = .006) and 60 minutes (43.2 [9.5] vs 48.4 [11.3] nmol/L; *P* = .011) after Synacthen injection (see Fig. 2). Mean [SD] salivary cortisone levels did not differ significantly between the COC and control groups at baseline (39.6 [10.8] vs 35.0 [11.5] nmol; *P* = .34), but was significantly lower in the COC group at 30 minutes (62.5 [14.3] vs 66.6 [15.0] nmol/L; *P* = .0498) and 60 minutes (78.0 [15.2] vs 83.8 [17.7] nmol/L; *P* = .035) after Synacthen injection (see Fig. 2).

The LRLs at 30 minutes after Synacthen injection were calculated for plasma cortisol (753 nmol/L for the COC group, 484 nmol/L for the control group), salivary cortisol (14.0 nmol/L for the COC group, 16.7 nmol/L for the control group), and salivary cortisone (34.4 nmol/L for the COC group, 37.0 nmol/L for the control group) (Table 1 and Fig. 2). At 60 minutes, the corresponding LRLs were 870 vs 566 nmol/L for plasma cortisol, 24.4 vs 26.2 nmol/L for salivary cortisol, and 48.1 vs 48.8 nmol/L for salivary cortisone (see Table 1 and Fig. 2). At 30 and 60 minutes after Synacthen, the LRL for salivary cortisol was 16% and 7% lower for the COC group than the controls, whereas the LRL for salivary cortisone was 7% and 1% lower for the COC group than the controls, respectively. Thus, the smallest difference in LRLs between the COC and the control groups was seen for salivary cortisone; however, these differences were not significant for either salivary cortisol or cortisone at 30 and 60 minutes when tested by bootstrap simulation.

**Table 1.** Lower reference limits at 30 and 60 minutes following short Synacthen test calculated by parametric methods

Measurand	Lower reference limit, nmol/L			
	30 min		60 min	
	COC group	Control group	COC group	Control group
Plasma cortisol	753 (659-847)*^a^*	484 (455-513)	870 (770-969)*^a^*	566 (537-596)
Salivary cortisol	14.0 (10.7-17.2)	16.7 (13.3-20.2)	24.4 (20.1-28.6)	26.2 (21.5-30.9)
Salivary cortisone	34.4 (28.2-40.7)	37.0 (30.8-43.2)	48.1 (41.3-54.8)	48.8 (41.5-56.2)

Data are given as mean (90% CI).

Abbreviation: COC, combined oral contraceptive.

^*a*^Significantly (*P* < .05) higher compared to control group.

Differences in ethinylestradiol dose in the COCs did not influence plasma cortisol, salivary cortisol, or salivary cortisone levels (Supplementary Fig. S1 (40)). In addition, we found no significant differences in salivary cortisol and salivary cortisone between users of COCs with different types of synthetic progestins. However, mean plasma cortisol at 60 minutes was significantly higher in the women using COCs containing dienogest compared to levonorgestrel, but without significant difference observed at other time points (Supplementary Fig. S2 (40)).

### Relation Between Cortisol and Cortisone in Plasma and Saliva

**Figure 3.**
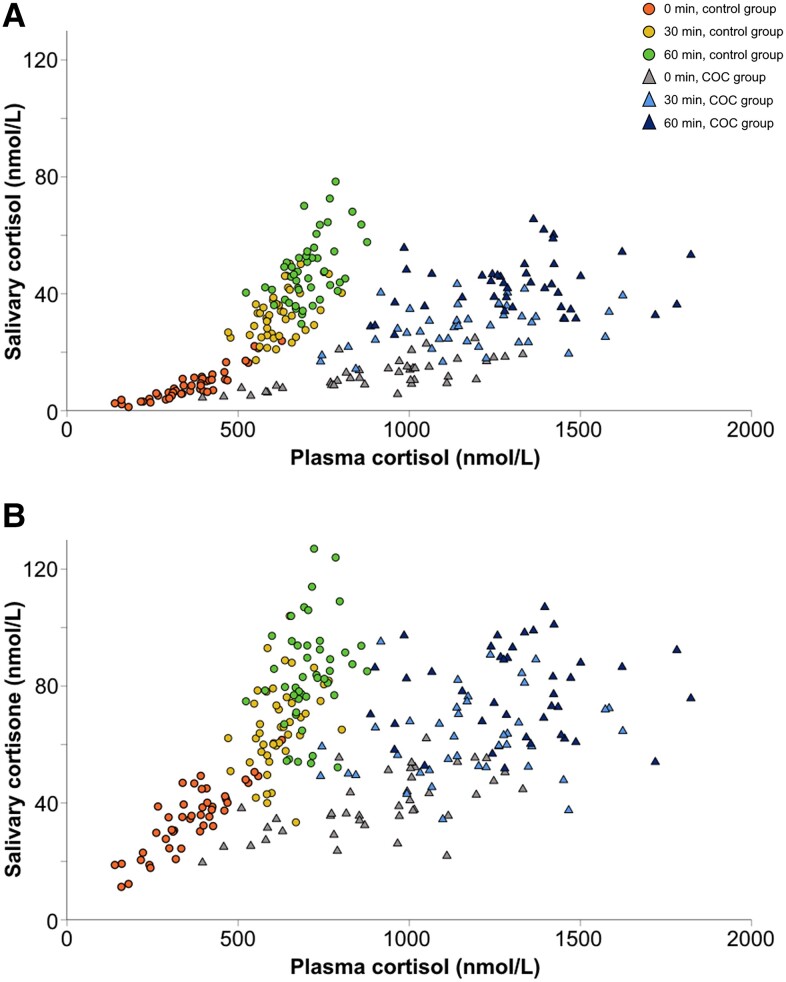
Pivot point around 500 nmol/L for plasma cortisol is seen for controls, where a sharp increase in salivary cortisol A, and salivary cortisone B, occurs. No pivot point was seen for the combined oral contraceptives (COC) group.

When plotting plasma cortisol vs salivary cortisol levels, a pivot point is seen around a plasma cortisol concentration of 500 nmol/L, after which both salivary cortisol and salivary cortisone increase more rapidly (Fig. 3). No corresponding pivot point was found for the COC group (see Fig. 3).

**Figure 4.**
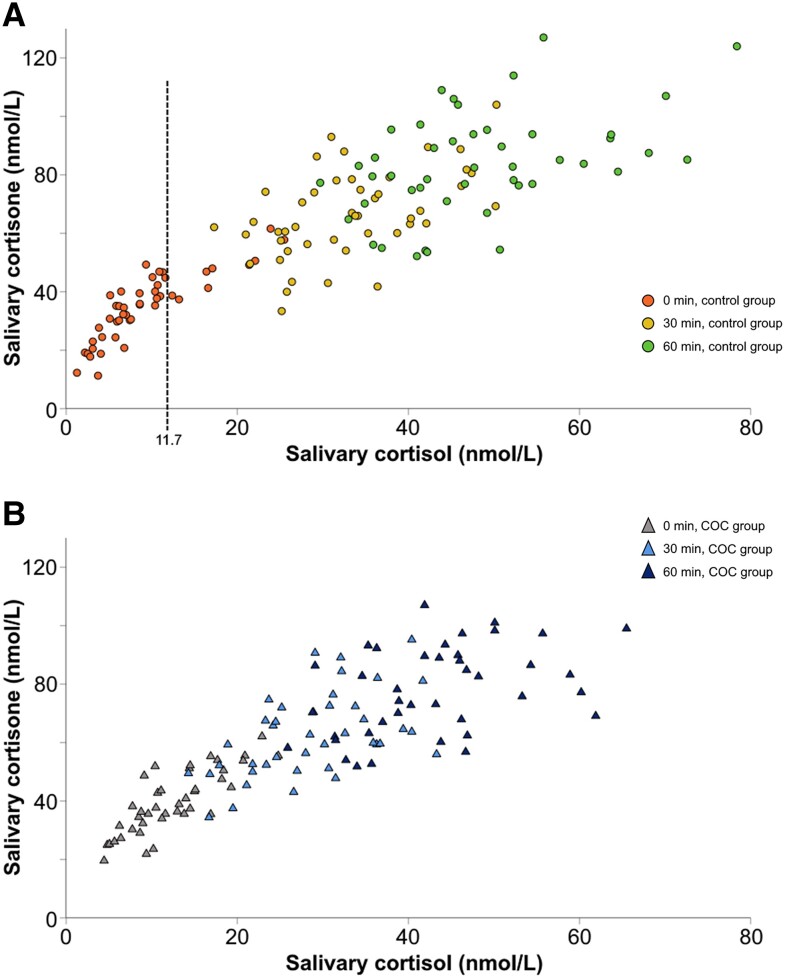
Salivary cortisol:cortisone relationship as concentrations increase. A pivot point of 11.7 nmol/L (dashed vertical line) for salivary cortisol is shown in the control group A, after which salivary cortisol increases more than salivary cortisone. No pivot point was found for the combined oral contraceptive (COC) group, B.

The mean cortisol:cortisone ratio in saliva was higher in the COC group compared to the control group at baseline (0.31 vs 0.24; *P* < .001), but was not significantly different at 30 (0.47 vs 0.51; *P* = .10) or 60 minutes (0.56 vs 0.59; *P* = .35). There was an increase in the cortisol:cortisone ratio with higher salivary cortisol concentrations. A pivot point where this increase in ratio occurred was calculated at a salivary cortisol concentration of 11.7 nmol/L, after which salivary cortisol increased steeper compared to salivary cortisone (Fig. 4). No such pivot point was seen for the COC group (see Fig. 4).

### Effect of Age, Baseline Concentrations, and Sampling Time Point

Increasing age was associated with decreasing plasma cortisol and salivary cortisol and cortisone at all time points during the SST, but was statistically significant only within the COC group (data not shown). Higher baseline concentrations were associated with a higher SST-stimulated concentration at 30 and 60 minutes for all measurands (see Fig. 1). Baseline cortisol and cortisone levels were higher in the control group earlier in the morning (07:00-09:00 hours) compared to later in the morning (09:00-11:00 hours): plasma cortisol (*P* < .001), salivary cortisol (*P* = .002), and salivary cortisone (*P* = .007). However, the differences were marginal in the COC group: plasma cortisol (*P* = .12), salivary cortisol (*P* = .023), and salivary cortisone (*P* = .059) (Supplementary Table S3 and Supplementary Fig. S3 (40)). No effect of time of day was observed for the SST-stimulated samples (see Supplementary Fig. S3 (40)).

## Discussion

The main finding of this study is that the LRLs for salivary cortisol and cortisone responses to the SST are minimally influenced by COC use. These LRLs may serve as tentative cutoffs to exclude AI. Notably, salivary cortisone after SST seems least affected by COC use.

The RIs for 30-minute and 60-minute samples during SST were calculated separately for the COC and control groups. According to the Clinical and Laboratory Standards Institute guidelines, RIs should be based on at least 39 valid observations, although 120 observations are desirable to establish robust RIs with 90% CIs (39). The Gaussian distribution of data within each group allowed us to calculate RIs using parametric methods. Due to a limited number of observations and prioritizing tight RIs to obtain high sensitivity for detecting AI, we chose to calculate RIs by the UMVUE method, which showed results similar to the bootstrap quantile estimator (data not shown). The validity of our calculated LRLs for salivary cortisol and cortisone is strengthened by the similarities to previously published cutoffs (25, 30, 36, 42). Thus, a salivary cortisone analyzed with LC-MS/MS above 37 nmol/L at 30 minutes after SST or above 49 nmol/L at 60 minutes is highly likely to exclude AI regardless of COC use. Likewise, AI could likely be excluded by salivary cortisol values above 17 nmol/L at 30 minutes and 26 nmol/L at 60 minutes, respectively. Further studies on salivary cortisol and cortisone during SST in patients with and without AI using COCs are warranted to evaluate diagnostic accuracy. The proposed cutoffs may be applicable to other LC-MS/MS methods after validation but may differ for immunoassays (43, 44).

The Endocrine Society guidelines recommend using the same serum/plasma cortisol cutoff at 30 and 60 minutes after SST. For salivary cortisol and cortisone, we suggest that different cutoffs be used at 30 and 60 minutes, as concentrations continue to increase considerably between these time points. Salivary cortisone may have an advantage over salivary cortisol as a diagnostic analyte, since it is considerably more robust against confounding factors including blood contamination, topical hydrocortisone, and licorice consumption (23, 27). Interestingly, recent data also show good diagnostic properties for salivary cortisone sampling on awakening as a screening test for AI (45, 46). However, as salivary cortisol is more widespread and also shows stability against CBG variations, the SST can still be reliably performed analyzing salivary cortisol.

Synacthen-stimulated salivary cortisol and cortisone concentrations were slightly lower in women using COCs compared to controls. Thus, applying a cutoff based on non–COC-using controls would not increase the risk of missing AI, which is an obvious risk for plasma cortisol measurements in this situation. A possible mechanism for lower SST-stimulated salivary cortisol and cortisone concentrations in the COC group relates to differences in CBG levels. In a high-estrogen state, newly released cortisol from the adrenal cortex will have more CBG-binding sites available to occupy before CBG saturation is reached and free cortisol rises more rapidly with subsequent increases of salivary cortisol and cortisone levels. This is in line with our results showing that the relative SST-stimulated increases of salivary cortisol and cortisone are larger than the relative increase in total plasma cortisol concentrations, and that the relative increase is larger in the control group compared to the COC group (see Supplementary Table S1 (40)). The relative increase for salivary cortisol is in line with previous studies, but there is a lack of earlier data for salivary cortisone (29, 31). The pivot point in the control group for a steeper relative increase in salivary cortisol and cortisone was seen at plasma cortisol levels around 500 nmol/L, suggesting this to be the point of CBG saturation (see Fig. 3) (25). After CBG saturation, free cortisol and subsequent salivary analytes increase relatively more compared to plasma cortisol, as seen in Supplementary Table S2 (40). However, the lack of such a pivot point and the less pronounced relative increases of salivary cortisol and cortisone for the COC group may be an effect of higher and possibly variable levels of CBG (see Fig. 3 and Supplementary Table S2 (40)).

The present study and previous data show higher salivary cortisol:cortisone ratios with increasing concentrations, indicating that higher levels of cortisol overwhelm the capacity of 11β-HSD2 in the salivary glands (23, 47). This is calculated to occur at salivary cortisol concentration of 11.7 nmol/L, which has not been presented previously (see Fig. 4). The use of salivary cortisone for diagnostic purposes has been questioned as 11β-HSD2 activity has been proposed to be increased by estrogen and synthetic progestins by some but not all earlier studies (48-50). The lack of a cortisol:cortisone ratio pivot point in the COC group indicates a possible hormonal effect on 11β-HSD2 (see Fig. 4).

The SSTs in this study were performed during the morning hours. As expected, baseline cortisol and cortisone levels were higher earlier in the morning, but the timing of the Synacthen injection did not influence concentrations of any of the analytes at the 30-minute or 60-minute time points, which is in line with previous studies (51). Due to the decrease in baseline values from 07:00 to 11:00 hours in this study, RIs for the baseline samples were not calculated. Within the present age span, we found a significant decrease of plasma cortisol, salivary cortisol, and salivary cortisone during the SST with increasing age. This finding is somewhat unexpected, and further studies of age effects and potential need for age-dependent cutoffs are warranted.

We found no significant dose-response effect from ethinylestradiol on plasma cortisol, salivary cortisol, or salivary cortisone concentrations over the dose range of the COCs studied (see Supplementary Fig. S1 (40)). This is in contrast to previous studies showing a dose-response effect on plasma CBG concentrations (5, 52), which might be due to few women using low and high ethinylestradiol doses in our study. Plasma cortisol at 60 minutes was significantly lower in women using COCs containing levonorgestrel compared to dienogest (see Supplementary Fig. S2 (40)), even though previous studies have not shown an effect on cortisol levels based on synthetic progestin type (6, 18). These results should, however, be interpreted cautiously due to low number of observations.

The strengths of our study include its prospective design with similar numbers of healthy women with and without commonly used types of ethinylestradiol-containing COCs and performing a standard SST, allowing the results to be applied in a clinical setting. To the best of our knowledge, this is the largest study of salivary cortisol and cortisone responses to an SST in women using COCs. To evaluate the diagnostic accuracy of salivary cortisol and cortisone for an SST in hyperestrogenemic women, subsequent studies also need to include a substantial number of patients with a clinical suspicion of AI. Finally, the presented cutoffs cannot immediately be extrapolated to other analytical methods for salivary steroids, other hyperestrogenemic states such as during pregnancy, states with low CBG levels such as cirrhosis, postmenopausal women, and men, although earlier studies have not indicated significant sex differences in glucocorticoid responses.

In conclusion, the diagnostic workup for AI with an SST in an ambulatory setting can be simplified for women using COCs by analyzing salivary cortisol and cortisone without the need for stopping COCs. Our work supports the use of the same cutoffs for COC users as for non-COC users.

## Data Availability

The data sets generated during and/or analyzed during the current study are available from the corresponding author on reasonable request.

## Abbreviations

11β-HSD2: 11β-hydroxysteroid dehydrogenase type 2
AI: adrenal insufficiency
CBG: corticosteroid-binding globulin
COC: combined oral contraceptive
CV: coefficient of variation
LC-MS/MS: liquid chromatography-tandem mass spectrometry
LRL: lower reference limit
RI: reference interval
SST: short Synacthen test
UMVUE: uniformly minimum variance unbiased estimator

## References

[1] Bergthorsdottir R, Leonsson-Zachrisson M, Odén A, Johannsson G. Premature mortality in patients with Addison's disease: a population-based study. J Clin Endocrinol Metab. 2006;91(12):4849‐4653. 16968806 10.1210/jc.2006-0076

[2] Burman P, Mattsson AF, Johannsson G, et al Deaths among adult patients with hypopituitarism: hypocortisolism during acute stress, and de novo malignant brain tumors contribute to an increased mortality. J Clin Endocrinol Metab. 2013;98(4):1466‐1475. 23457412 10.1210/jc.2012-4059

[3] Ho W, Druce M. Quality of life in patients with adrenal disease: a systematic review. Clin Endocrinol (Oxf). 2018;89(2):119‐128. 29672878 10.1111/cen.13719

[4] Wood JB, Frankland AW, James VH, Landon J. A rapid test of adrenocortical function. Lancet. 1965;1(7379):243‐245. 14238068 10.1016/s0140-6736(65)91526-6

[5] Charmandari E, Nicolaides NC, Chrousos GP. Adrenal insufficiency. Lancet. 2014;383(9935):2152‐2167. 24503135 10.1016/S0140-6736(13)61684-0

[6] Musa BU, Seal US, Doe RP. Elevation of certain plasma proteins in man following estrogen administration: a dose-response relationship. J Clin Endocrinol Metab. 1965;25(9):1163‐1166. 4284083 10.1210/jcem-25-9-1163

[7] El-Farhan N, Pickett A, Ducroq D, et al Method-specific serum cortisol responses to the adrenocorticotrophin test: comparison of gas chromatography-mass spectrometry and five automated immunoassays. Clin Endocrinol (Oxf). 2013;78(5):673‐680. 22994849 10.1111/cen.12039

[8] Hawley JM, Owen LJ, Lockhart SJ, et al Serum cortisol: an up-to-date assessment of routine assay performance. Clin Chem. 2016;62(9):1220‐1229. 27440512 10.1373/clinchem.2016.255034

[9] Meulenberg PM, Ross HA, Swinkels LM, Benraad TJ. The effect of oral contraceptives on plasma-free and salivary cortisol and cortisone. Clin Chim Acta. 1987;165(2-3):379‐385. 3652459 10.1016/0009-8981(87)90183-5

[10] Sandberg AA, Slaunwhite WR Jr. Transcortin: a corticosteroid-binding protein of plasma. II. Levels in various conditions and the effects of estrogens. J Clin Invest. 1959;38(8):1290‐1297. 13673085 10.1172/JCI103904 PMC442083

[11] Kawagoe S, Hiroi M. Effects of several estrogenic compounds on serum corticosteroid-binding globulin (CBG) and the estrogen thresholds for increasing CBG. Nihon Naibunpi Gakkai Zasshi. 1978;54(5):666‐675. 211055 10.1507/endocrine1927.54.5_666

[12] Moore DE, Kawagoe S, Davajan V, Mishell DR, Nakamura RM. An in vivo system in man for quantitation of estrogenicity. I. Physiologic changes in binding capacity of serum corticosteroid-binding globulin. Am J Obstet Gynecol. 1978;130(4):475‐481. 629295 10.1016/0002-9378(78)90293-4

[13] Wiegratz I, Jung-Hoffmann C, Kuhl H. Effect of two oral contraceptives containing ethinylestradiol and gestodene or norgestimate upon androgen parameters and serum binding proteins. Contraception. 1995;51(6):341‐346. 7554973 10.1016/0010-7824(95)00098-u

[14] Qureshi AC, Bahri A, Breen LA, et al The influence of the route of oestrogen administration on serum levels of cortisol-binding globulin and total cortisol. Clin Endocrinol. 2007;66(5):632‐635. 10.1111/j.1365-2265.2007.02784.x 17492949

[15] Jung-Hoffmann C, Storch A, Kuhl H. Serum concentrations of ethinylestradiol, 3-keto-desogestrel, SHBG, CBG and gonadotropins during treatment with a biphasic oral contraceptive containing desogestrel. Horm Res. 1992;38(3-4):184‐189. 1306851 10.1159/000182537

[16] Díaz S, Pavez M, Brandeis A, Cárdenas H, Croxatto HB. A longitudinal study on cortisol, prolactin and thyroid hormones in users of Norplant subdermal implants or a copper T device. Contraception. 1989;40(4):505‐517. 2510969 10.1016/0010-7824(89)90056-5

[17] Fahmy K, el-Gazar A, Eisa I, Ghonaim M, Saad S, Afifi A. Levels of serum steroid hormones in intrauterine contraceptive device users. Gynecol Endocrinol. 1991;5(1):1‐5. 1897380 10.3109/09513599109049936

[18] Sobbrio GA, Granata A, Granese D, et al Sex hormone binding globulin, cortisol binding globulin, thyroxine binding globulin, ceruloplasmin: changes in treatment with two oral contraceptives low in oestrogen. Clin Exp Obstet Gynecol. 1991;18(1):43‐45. 1829029

[19] Panton KK, Mikkelsen G, Irgens WØ, et al New reference intervals for cortisol, cortisol binding globulin and free cortisol index in women using ethinyl estradiol. Scand J Clin Lab Invest. 2019;79(5):314‐319. 31161807 10.1080/00365513.2019.1622031

[20] Perogamvros I, Keevil BG, Ray DW, Trainer PJ. Salivary cortisone is a potential biomarker for serum free cortisol. J Clin Endocrinol Metabol. 2010;95(11):4951‐4958. 10.1210/jc.2010-1215 20685855

[21] Blair J, Adaway J, Keevil B, Ross R. Salivary cortisol and cortisone in the clinical setting. Curr Opin Endocrinol Diabetes Obes. 2017;24(3):161‐168. 28375882 10.1097/MED.0000000000000328

[22] Coelli S, Farias CB, Soares AA, et al Influence of age, gender and body mass index on late-night salivary cortisol in healthy adults. Clin Chem Lab Med. 2017;55(12):1954‐1961. 28593924 10.1515/cclm-2016-1100

[23] Bäcklund N, Brattsand G, Israelsson M, et al Reference intervals of salivary cortisol and cortisone and their diagnostic accuracy in Cushing's syndrome. Eur J Endocrinol. 2020;182(6):569‐582. 32213657 10.1530/EJE-19-0872

[24] Laudat MH, Cerdas S, Fournier C, Guiban D, Guilhaume B, Luton JP. Salivary cortisol measurement: a practical approach to assess pituitary-adrenal function. J Clin Endocrinol Metab. 1988;66(2):343‐348. 2828410 10.1210/jcem-66-2-343

[25] Cornes MP, Ashby HL, Khalid Y, Buch HN, Ford C, Gama R. Salivary cortisol and cortisone responses to tetracosactrin (synacthen). Ann Clin Biochem. 2015;52(5):606‐610. 25724424 10.1177/0004563215577838

[26] Perogamvros I, Owen LJ, Newell-Price J, Ray DW, Trainer PJ, Keevil BG. Simultaneous measurement of cortisol and cortisone in human saliva using liquid chromatography-tandem mass spectrometry: application in basal and stimulated conditions. J Chromatogr B Analyt Technol Biomed Life Sci. 2009;877(29):3771‐3775. 10.1016/j.jchromb.2009.09.014 19783236

[27] Imamovic M, Bäcklund N, Lundstedt S, et al Confounding effects of liquorice, hydrocortisone, and blood contamination on salivary cortisol but not cortisone. Endocr Connect. 2022;12(1):e220324. 36383173 10.1530/EC-22-0324 PMC9782436

[28] Debono M, Harrison RF, Whitaker MJ, et al Salivary cortisone reflects cortisol exposure under physiological conditions and after hydrocortisone. J Clin Endocrinol Metab. 2016;101(4):1469‐1477. 26812690 10.1210/jc.2015-3694

[29] Aardal-Eriksson E, Karlberg BE, Holm AC. Salivary cortisol: an alternative to serum cortisol determinations in dynamic function tests. Clin Chem Lab Med. 1998;36(4):215‐222. 9638346 10.1515/CCLM.1998.037

[30] Dušková M, Šimůnková K, Vítků J, et al A comparison of salivary steroid levels during diagnostic tests for adrenal insufficiency. Prague Med Rep. 2016;117(1):18‐33. 10.14712/23362936.2016.2 26995200

[31] Perogamvros I, Owen LJ, Keevil BG, Brabant G, Trainer PJ. Measurement of salivary cortisol with liquid chromatography-tandem mass spectrometry in patients undergoing dynamic endocrine testing. Clin Endocrinol. 2010;72(1):17‐21. 10.1111/j.1365-2265.2009.03582.x 19302583

[32] Marcus-Perlman Y, Tordjman K, Greenman Y, et al Low-dose ACTH (1 µg) salivary test: a potential alternative to the classical blood test. Clin Endocrinol (Oxf). 2006;64(2):215‐218. 16430723 10.1111/j.1365-2265.2006.02451.x

[33] Raff H . Utility of salivary cortisol measurements in Cushing's syndrome and adrenal insufficiency. J Clin Endocrinol Metab. 2009;94(10):3647‐3655. 19602555 10.1210/jc.2009-1166

[34] Tan SYT, Tan HC, Zhu L, et al Expanding the use of salivary cortisol as a non-invasive outpatient test in the dynamic evaluation of suspected adrenal insufficiency. Endocr Connect. 2023;12(4):e230004. 36799247 10.1530/EC-23-0004 PMC10083658

[35] Mak IYF, Au Yeung BYT, Ng YW, et al Salivary cortisol and cortisone after low-dose corticotropin stimulation in the diagnosis of adrenal insufficiency. J Endocr Soc. 2017;1(2):96‐108. 29264470 10.1210/js.2016-1056 PMC5686556

[36] Deutschbein T, Unger N, Mann K, Petersenn S. Diagnosis of secondary adrenal insufficiency in patients with hypothalamic-pituitary disease: comparison between serum and salivary cortisol during the high-dose short synacthen test. Eur J Endocrinol. 2009;160(1):9‐16. 18952762 10.1530/EJE-08-0600

[37] Deutschbein T, Broecker-Preuss M, Flitsch J, et al Salivary cortisol as a diagnostic tool for Cushing's syndrome and adrenal insufficiency: improved screening by an automatic immunoassay. Eur J Endocrinol. 2012;166(4):613‐618. 22214924 10.1530/EJE-11-0945

[38] Israelsson M, Brattsand R, Brattsand G. 20α- and 20β-dihydrocortisone may interfere in LC-MS/MS determination of cortisol in saliva and urine. Ann Clin Biochem. 2018;55(3):341‐347. 28726485 10.1177/0004563217724178

[39] Horowitz GL, Altaie S, Boyd JC, et al Defining, Establishing, and Verifying Reference Intervals in the Clinical Laboratory; Approved Guideline—Third Edition. Clinical and Laboratory Standards Institute; 2010.

[40] Bäcklund N, Lundstedt S, Tornevi A, et al Data from: Salivary cortisol and cortisone can circumvent the confounding effects of combined oral contraceptives in the short Synacthen test. Figshare. Deposited January 6, 2024. Doi: 10.6084/m9.figshare.24947640.v2 PMC11180507 38173358

[41] Raff H, Singh RJ. Measurement of late-night salivary cortisol and cortisone by LC-MS/MS to assess preanalytical sample contamination with topical hydrocortisone. Clin Chem. 2012;58(5):947‐948. 22377529 10.1373/clinchem.2012.182717

[42] Kalaria T, Agarwal M, Kaur S, et al Hypothalamic-pituitary-adrenal axis suppression: the value of salivary cortisol and cortisone in assessing hypothalamic-pituitary-adrenal recovery. Ann Clin Biochem. 2020;57(6):456‐460. 32961064 10.1177/0004563220961745

[43] Bäcklund N, Brattsand G, Lundstedt S, et al Salivary cortisol and cortisone in diagnosis of Cushing's syndrome: a comparison of six different analytical methods. Clin Chem Lab Med. 2023;61(10):1780‐1791. 37013440 10.1515/cclm-2023-0141

[44] Javorsky BR, Raff H, Carroll TB, et al New cutoffs for the biochemical diagnosis of adrenal insufficiency after ACTH stimulation using specific cortisol assays. J Endocr Soc. 2021;5(4):bvab022. 33768189 10.1210/jendso/bvab022 PMC7975762

[45] Debono M, Elder CJ, Lewis J, et al Home waking salivary cortisone to screen for adrenal insufficiency. NEJM Evid. 2023;2(2). Doi: 10.1056/EVIDoa2200182 38320034

[46] Elder CJ, Harrison RF, Cross AS, et al Use of salivary cortisol and cortisone in the high- and low-dose synacthen test. Clin Endocrinol (Oxf). 2018;88(6):772‐778. 29106701 10.1111/cen.13509

[47] Palermo M, Shackleton CH, Mantero F, Stewart PM. Urinary free cortisone and the assessment of 11 β-hydroxysteroid dehydrogenase activity in man. Clin Endocrinol (Oxf). 1996;45(5):605‐611. 8977758 10.1046/j.1365-2265.1996.00853.x

[48] Darnel AD, Archer TK, Yang K. Regulation of 11β-hydroxysteroid dehydrogenase type 2 by steroid hormones and epidermal growth factor in the Ishikawa human endometrial cell line. J Steroid Biochem Mol Biol. 1999;70(4-6):203‐210. 10622409 10.1016/s0960-0760(99)00116-8

[49] Sun K, Yang K, Challis JR. Regulation of 11β-hydroxysteroid dehydrogenase type 2 by progesterone, estrogen, and the cyclic adenosine 5'-monophosphate pathway in cultured human placental and chorionic trophoblasts. Biol Reprod. 1998;58(6):1379‐1384. 9623596 10.1095/biolreprod58.6.1379

[50] Chapman K, Holmes M, Seckl J. 11β-hydroxysteroid dehydrogenases: intracellular gate-keepers of tissue glucocorticoid action. Physiol Rev. 2013;93(3):1139‐1206. 23899562 10.1152/physrev.00020.2012 PMC3962546

[51] Munro V, Elnenaei M, Doucette S, Clarke DB, Imran SA. The effect of time of day testing and utility of 30 and 60 minute cortisol values in the 250 mcg ACTH stimulation test. Clin Biochem. 2018;54:37‐41. 29458002 10.1016/j.clinbiochem.2018.02.010

[52] Schwartz U, Hammerstein J. The oestrogenic potency of various contraceptive steroids as determined by their effects on transcortin-binding capacity. Acta Endocrinol (Copenh). 1974;76(1):159‐171. 4406579 10.1530/acta.0.0760159

